# A population pharmacokinetic model for individualised dosage regimens of vancomycin in Chinese neonates and young infants

**DOI:** 10.18632/oncotarget.22114

**Published:** 2017-10-26

**Authors:** Lin Song, Cui-Yao He, Nan-Ge Yin, Fang Liu, Yun-Tao Jia, Yao Liu

**Affiliations:** ^1^ Department of Pharmacy, Ministry of Education Key Laboratory of Child Development and Disorders, China International Science and Technology Cooperation Base of Child Development and Critical Disorders, Chongqing Key Laboratory of Pediatrics, Children's Hospital of Chongqing Medical University, Chongqing 400014, China; ^2^ Department of Pharmacy, Southwest Hospital, Third Military Medical University, Chongqing 400038, China

**Keywords:** vancomycin, Chinese young infants, population pharmacokinetics, phoenix NLME

## Abstract

Population pharmacokinetic (PPK) modelling is an easy and impartment method for estimating drug concentration for use inindividualized therapy, especially for young patients and to help protect drug-induced diseases. The purpose of this study was to develop a PPK model for effective dosing of vancomycin in Chinese neonates and young infants. The PPK modelling tool Phoenix^®^ NLME^™^ was use to assess demographic and routine clinical pharmacokinetic (PK) data retrospectively collected for patients admitted to Children's Hospital of Chongqing Medical University between 2011 and 2016. Data of patients admitted to the hospital between January and June of 2017 were used in validation study, and the final model was also preliminary validated in 2 cases in another hospital. A total of 421 serum samples from 316 patients were included in the initial PPK analysis. A two-compartment PPK model was developed, and exponential-error model was used to describe inter-individual variability of clearance. Residual variability was described by an additive model. The final PPK model was demonstrated as valid by internal and external model evaluation. Of note, the clearance and volume of vancomycin in Chinese neonates and young infants may be greater than in Caucasians. Herein, we describe the establishment of an accurate PPK model of vancomycin for Chinese neonates and young infants, which may be useful as a dosing algorithm for this particular paediatric population.

## INTRODUCTION

Despite approximately 6 decades of clinical use, vancomycin has remained a consistently effective antibacterial agent for the treatment of serious Gram-positive infections involving meticillin (*al*. methicillin)-resistant *Staphylococcus aureus* (MRSA) [1]. It is also increasingly used in neonates, with up to 10% of neonatal intensive care unit patients receiving at least one dose [2]. More recently, vancomycin has become one of the most studied antibiotics because of its toxicity, resulting in serum vancomycin concentrations being established for therapeutic drug monitoring (TDM) to maintain its efficacy and minimize the risk of ear and renal toxicity [3, 4].

Among the population pharmacokinetic (PPK) studies for predicting vancomycin concentrations for paediatric clinical use, over 10 have involved Caucasians and only 1 has involved Asians. That single study on Asian paediatric patients focused on premature neonates in Malaysia [5]. Thus, there is a lacuna in the literature of pharmacokinetic (PK) evaluation of vancomycin, precluding the availability of a dosing algorithm for individualized therapy, in Asian populations in general and in Chinese neonates and young infants in particular.

While the published studies have identified growth and maturational changes as significant covariates in describing variability in vancomycin clearance (CL) [1], they have not defined the markers of renal function, such as serum creatinine (Cr) and glomerular filtration rate (GFR), that may contribute to establishment of an accurate dosing algorithm for neonates and young infants [6–8]. Although such parameters are demonstrated covariates in vancomycin dosing algorithms for adults, their role in estimation of vancomycin CL in neonates and young infants remains unclear [9, 10]. In addition, the existing PPK models were mostly established with small sample sizes, which severely limits their clinical applicability and application in individualised dosage regimens [1, 11].

Thus, the objective of this study was to develop a PPK model of vancomycin for use in Chinese neonates and young infants by using a relatively large sample size of patients, in order to obtain a robust PK dataset and establish an effective dosing algorithm of vancomycin in Chinese neonates and young infants.

## RESULTS

### Demographic and clinical characteristics of the study participants

**Figure 1:**
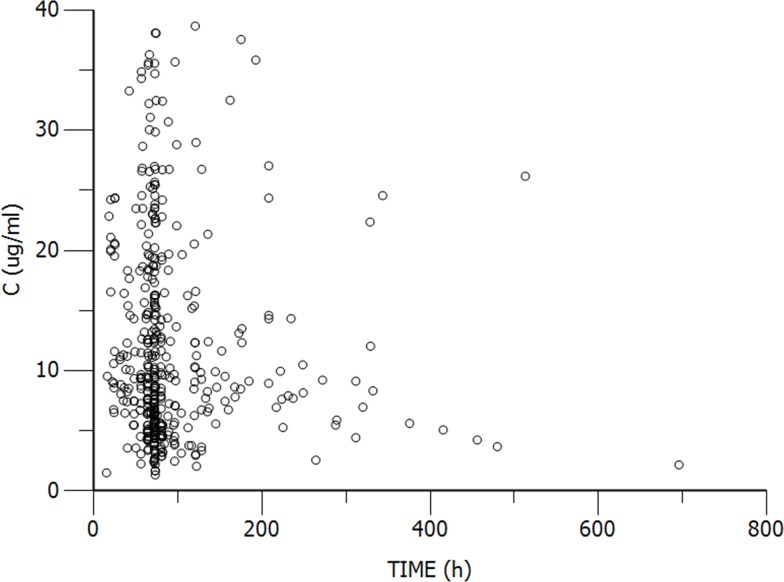
Scatter plots of observed concentration versus sample time, representing the total 421 samples from 316 patients

The study group of neonates and young infants represented a total of 421 samples from 316 patients that were included in the population PK analysis. Among the 316 patients, results of pathogenic bacteria cultures in 137 cases showed gram-positive bacterial infections, with 59 from blood cultures, and others from sputum, fester, and puncture fluid etc. For the remaining 179 patients, results of sputum, blood or other biological sample cultures were negative, and a few patients admitted before 2012 with no bacterial culture. There were marked differences in subject demographics (Table 1). The daily dose of vancomycinranged from 13.7-73.5 mg/kg, administered 2 to 4 times. The patients had serum concentration data for 1-6 time points. The distribution ranges of concentration and sampling time (time after first dose) were wide (Figure 1).

**Table 1:** Demographic and clinical characteristics of the study participants

Characteristic	Original group	External evaluation group
Patient total	316 (201/115)	19 (12/7)
Premature patients	102 (58/44)	7 (4/3)
Observations	421 (277/144)	27 (16/9)
Postnatal age at admission, d	24 (0, 60)	12 (3, 28)
Gestational age, w	37 (28, 41)	37 (28, 40)
Postnatal age at vancomycin determined, d	29 (2, 77)	9 (2, 35)
Birth body weight, kg	3.22 (1.25, 5.38)	3.25 (2.6, 4.85)
Body weight, kg	3.95 (1.25, 7.62)	4.09 (1.5, 5.15)
Height, cm	49 (35, 62.3)	47 (39, 59.8)
Scr, μmol/L	28.6 (12, 151)	30.2 (18.2, 78.6)
BUN, mmol/L	2.35 (0.48, 7.54)	2.88 (0.4, 6.79)
ALT, U/L	25.7 (5.4, 130.9)	23.3 (3.6, 45.8)
ALB, g/L	33.1 (13.5, 52.6)	34.3 (26, 49.3)
Panipenem treatment	168 (106/62)	12 (7/5)
Furosemide treatment	74 (39/35)	9 (5/4)
Vancomycin dose, mg/kg/d	36.7 (13.7, 73.5)	33.3 (20.6, 49.3)
Drug concentration, μg/mL	9.33 (1.34, 38.65)	9.54 (1.92, 28.06)

Data are presented as n (male/female) or median (minimum, maximum).

### PPK model

Preliminary analysis for the base model showed that the OFVs for exploration of the mean structure of the one- and two-compartment models were 2764.31 and 2623.54, respectively. These values were determined by taking into consideration the diagnostic scatter plots and inter-individual variability for the associated PK parameters. The two-compartment model resulted in a better fit for describing vancomycin concentrations in the study group of Chinese neonates and infants, while the additive model best described the residual variability. The shrinkage factors of V1, V2 and Q were all > 0.5, indicating minor inter-individual variability of the parameters that could be eliminated without significantly altering the parameter values or OFV; as such, each were excluded in the model building process.

In the modelling process, CL was validated by a hypothesis test using forward-inclusion and backward-elimination (Table 2). Each of the candidate covariates was analysed, with the concomitant drug therapy covariate represented by panipenem and furosemide. The final model was as follows:*V1*(l)=1.27, *V2*(l)=2.422, *CL*(l/h)=0.42^*^(*BBW*/3.22)^0.888^*^(*PNA*/29)^0.449^*^exp(*η_CL_*) and *Q*(l/h)=1.161, where 3.22 (kg) is the median BBW and the median PNA was29 (days). Thus, the median CL and Vd (volume of distribution, sum of V1 and V2) values of vancomycin in the Chinese neonates and young infants were about 0.106 l/h/kg (0.42 l/h divided by median weight of 3.95 kg) and 0.935 l/kg (3.692 l (sum of V1 and V2) divided by median weight of 3.95 kg).

**Table 2:** Stepwise and statistical values used for discrimination

Model no.^*^	Model description	OFV	ΔOFV	*P*
Forward-inclusion				
1	Basic model	2623.54		
2	Add PNA in model 1	2509.82	−113.72	< 0.01
3	Add PMA in model 2	2502.56	−7.26	< 0.01
4	Add BBW in model 3	2488.94	−13.62	< 0.01
Backward-elimination				
5	Remove PNA from model 4	2508.29	19.35	< 0.001
6	Remove PMA from model 4	2493.65	4.71	> 0.001
7	Remove BBW from model 4	2502.56	13.62	< 0.001
8	Remove PNA from model 6	2568.66	75.01	< 0.001
9	Remove BBW from model 6	2509.82	16.17	< 0.001

^*^Only the significant computational steps for the selection of covariates are shown.

### Model evaluation

#### Internal model evaluation

**Figure 2:**
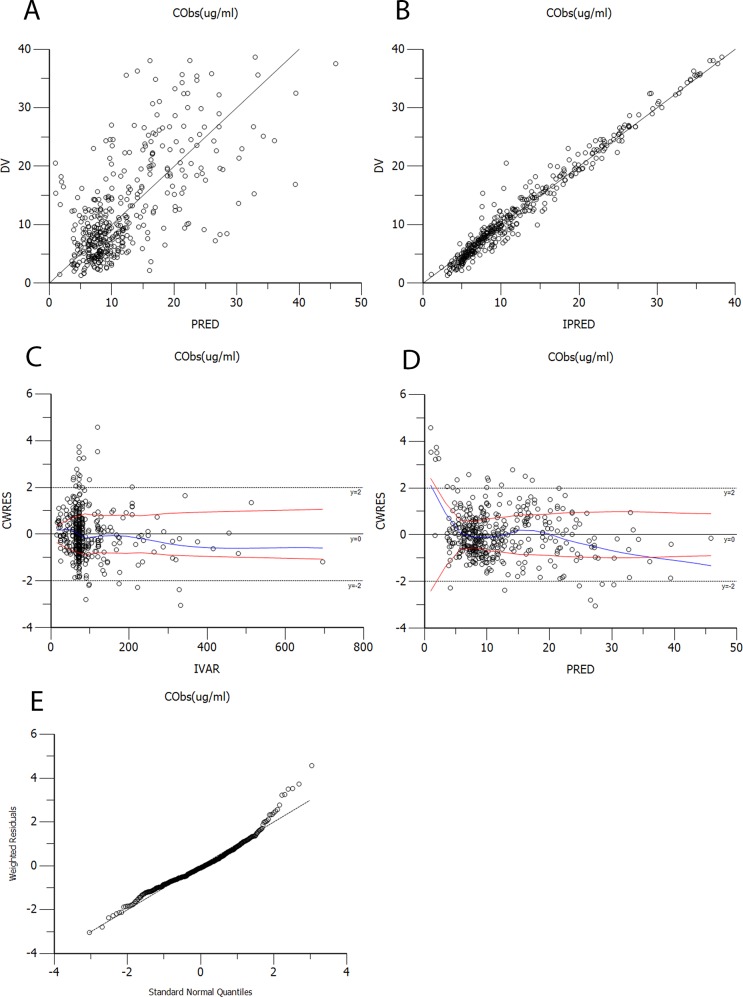
Diagnostic scatter plots of the base model **(A)** Observed versus population-predicted concentrations (DV vs. PRED); **(B)** Observed versus individual-predicted concentrations (DV vs. IPRED); **(C)** Conditional weighted residuals versus time (CWRES vs. IVAR); **(D)** Conditional weighted residuals versus population-predicted concentrations (CWRES vs. PRED); **(E)** Quantile-quantile plot of the components of conditional weighted residuals.

**Figure 3:**
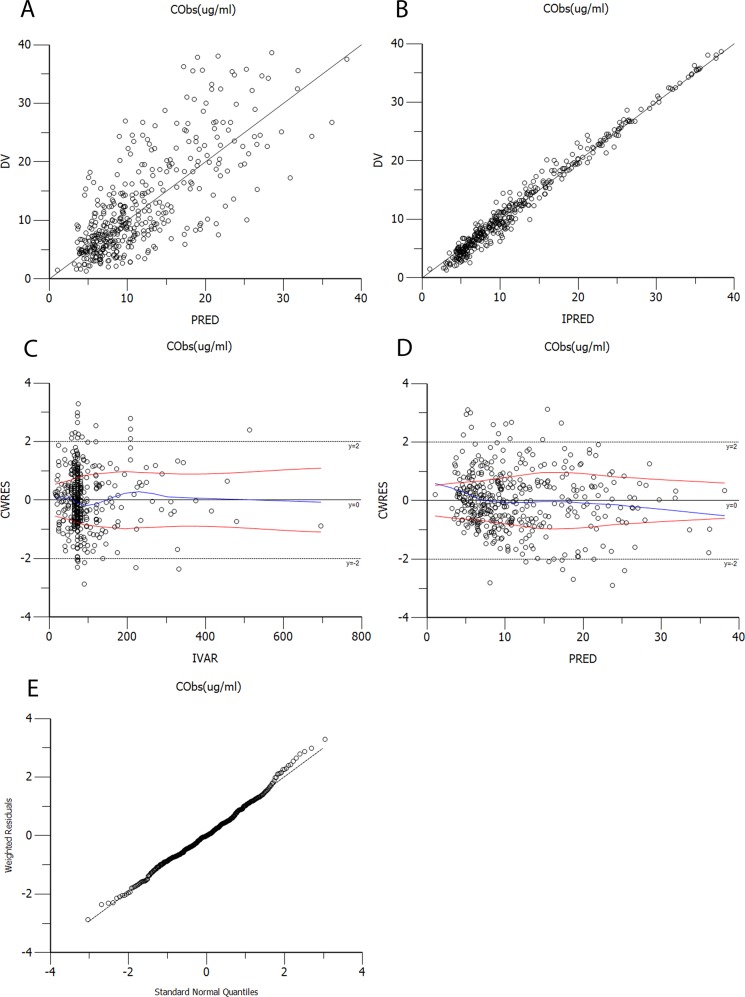
Diagnostic scatter plots of the final model **(A)** Observed versus population-predicted concentrations (DV vs. PRED); **(B)** Observed versus individual-predicted concentrations (DV vs. IPRED); **(C)** Conditional weighted residuals versus time (CWRES vs. IVAR); **(D)** Conditional weighted residuals versus population-predicted concentrations (CWRES vs. PRED); **(E)** Quantile-quantile plot of the components of conditional weighted residuals.

**Figure 4:**
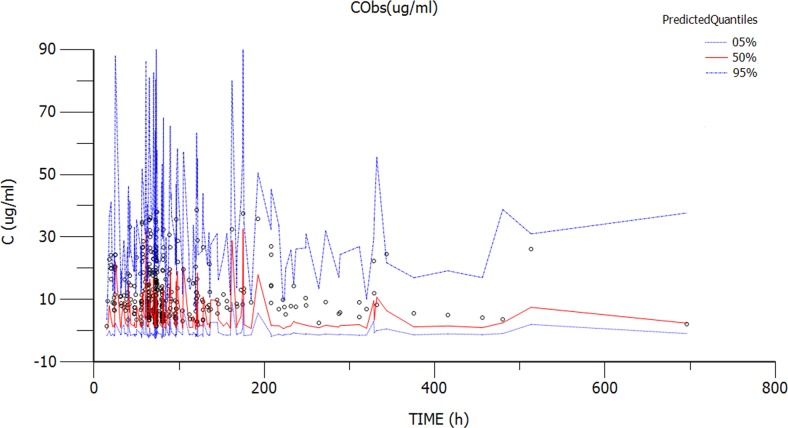
Visual predictive check obtained from 1000 simulations of the database Dotted lines indicate the nonparametric 5th to 95th intervals of the simulations, whereas the points indicate the observed concentrations.

The diagnostic scatter plots of the base model and the final model are shown in Figures 2 and 3, respectively. The quantile-quantile plot of the final model, which provided the best fit for the prediction of vancomycin concentrations, showed that the components of CWRES were normally distributed. The estimated covariates of the final model and 2,000 bootstrap replicates for vancomycin (Table 3) indicated a qualified stability for the final model. The VPC of the final model demonstrated that the DV concentration data were distributed within the 5th to 95th prediction interval approximately (Figure 4), supporting the precise performance of the final model.

**Table 3:** Final population pharmacokinetic parameter estimates of vancomycin and bootstrap validation

Parameter	Final model			Bootstrap		
	Estimate	SE	95% CI	Median	SE	95% CI
tvV1 (l)	1.27	0.191	0.895-1.644	1.255	0.247	0.771-1.739
tvV2 (l)	2.422	0.425	1.586-3.258	2.386	0.482	1.441-3.331
tvCL (l/h)	0.42	0.0124	0.395-0.444	0.416	0.0183	0.380-0.452
tvQ (l/h)	1.161	0.177	0.814-1.509	1.159	0.204	0.759-1.56
dCldBBW	0.888	0.12	0.652-1.124	0.885	0.15	0.591-1.179
dCldPNA	0.449	0.058	0.336-0.563	0.453	0.068	0.32-0.587
stdev0	2.187	0.194	1.807-2.568	2.158	0.232	1.704-2.613
ωCL	0.317	0.015	0.288-0.346	0.316	0.019	0.278-0.353

tvV1 = typical value of V1; tvV2 = typical value of V2; tvCL = typical value of CL; tvQ = typical value of Q; dCldBBW = fixed parameter coefficient of birth body weight; dCldPNA = fixed parameter coefficient of postnatal age; stdev0 = standard deviation; ωCL = variance of the inter-individual variability of CL.

#### External evaluation

**Figure 5:**
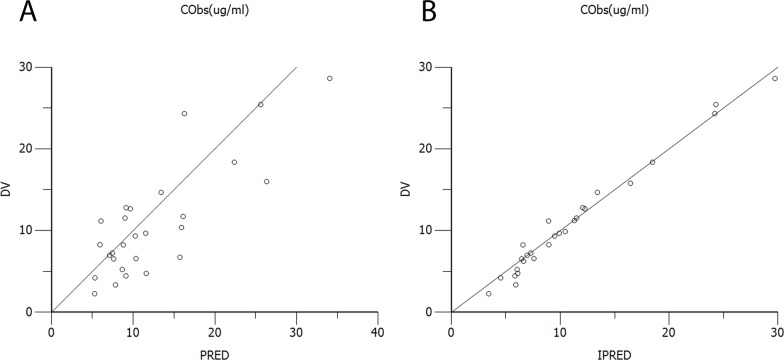
Plots of the external evaluation group **(A)** Observed versus population-predicted concentrations (DV vs. PRED); **(B)** Observed versus individual-predicted concentrations (DV vs. IPRED).

A total of 27 samples from 19 patients were used for the external evaluation of the final model. Population- and individual-predicted concentrations versus DV concentrations are shown in Figure 5, with differences ranging from −0.2% to 15%. MPE, MAE and MSPE were −0.29 ±0.99 ng/mL, 1.388±0.71 ng/mL and 1.928±1.665 ng/mL, respectively. Thus, the final model provided good prediction with low bias.

### Preliminary validation in another hospital

The established PPK model was preliminary validated in Southwest Hospital of the Third Military Medical University with 2 neonatal patients, providing limited available data at present. With estimates of the model fixed, the Phoenix^®^ NLME^TM^ tool allows for importing of patient demographic and medication data to estimate the steady trough concentration of the patient, and the predicted concentration was then compared with the TDM result. The 2 pneumonia neonatal patients with BBW of 3.9 kg and 4.5 kg, and blood cultures indicated the presence of *S. epidermidis* and *S. aureus*, respectively. Vancomycin was prescribed as 35 mg q8h, and 60 mg q8h, by the physicians. The blood sampling took place both before the 6th administration, when the patient was 23-day-old, and 25-day-old, respectively. The TDM report card showed that the vancomycin trough concentration was 4.26 μg/mL and 9.03 μg/mL. By applying the patients’ demographic data and medication information to the final PPK model, the predicted trough concentrations were 3.79 μg/mL and 8.73 μg/mL with 11.0% and 3.32%prediction error, respectively.

## DISCUSSION

The relationship between serum concentrations of vancomycin and treatment results in serious *S. aureus* infections is well established, and it is recommended that trough serum vancomycin concentrations be maintained at >10 μg/mL to avoid the development of resistance [12]. However, due to PK and clinical heterogeneities among neonates and young infants, it is difficult to achieve this target concentration with routine starting doses among this distinctive patient population [13]. PPK modelling may be a useful approach for developing an accurate and effective dosing algorithm to overcome this important clinical challenge [11, 14].

In this study, we used the population modelling approach to described the vancomycin PKs in Chinese neonates and young infants, which allowed for a quantitative systematic evaluation of the effect of related pathophysiological and clinical covariates. BBW and PNA were identified as the significant covariates for vancomycin CL. It is important to note that the median CL and Vd values of vancomycin in the Chinese neonates and young infants of this study were greater than previously reported values in Caucasian neonates and young infants [1] and Malaysian premature neonates [5]; certainly, more research, especially from multi-centre studies, is needed to confirm our findings and to uncover the underlying racial factors.

Vancomycin is eliminated almost exclusively by glomerular filtration [15]; therefore, variability of CL within and between neonates and young infants is likely correlated with the functional maturation of kidney or with SCr, which is related to the glomerular filtration rate; the latter, in particular, would agree with the observation that vancomycin CL in adults is closely related to SCr [1]. Of the 12 previous PK studies of vancomycin in neonates and young infants, 4 identified SCr concentration as significant for estimation of CL and dosage optimization of vancomycin [16–19], while the other 8 identified only growth and maturational factors, such as weight and age, as important [1, 5, 7]. Only one of those 12 studies identified BBW as a significant covariate influencing the CL of vancomycin [7]; moreover, this represented the largest of the studies—involving 273 preterm neonates—and its findings were consistent with ours.

In our study, 32% of the patients were preterm neonates. Since both BBW and GA influence kidney growth and function in infancy [20–22], a dosing algorithm that is based on BBW and PNA, as concluded in our study, is reasonable. Since SCr is known to be influenced by age, sex, muscle mass and diet [23]—limiting its utility as a marker of the glomerular filtration rate, especially for neonates [9, 24–26]—it was excluded from our modelling process; this practice is common in the related studies. Although some previous studies identified weight as a significant parameter that related to CL of vancomycin [1, 5, 7], our study showed birth body weight combined with PNA was preferable in Chinese neonates and young infants with our data, as weight is partly correlated to PNA. Our modelling did, however, consider concomitant therapies (i.e. panipenem and furosemide) as categorical covariates, but the factors produced no significant impact on the final model.

Complicated infections, including endocarditis, osteomyelitis, meningitis, and hospital-acquired, health care-associated or ventilator-associated pneumonia, require different trough serum vancomycin concentrations; relevant guidelines recommend 15-20 μg/mL or > 10 mg/L for other indications [14, 26]. For children, in particular, concentrations of 10-15 μg/mL are recommended [27], but studies have shown that the proportion of patients who reach therapeutic trough levels with the current empiric doses are < 40% [28, 29]; this rate is even lower in China, being 28.17% [30]. In our collected data, 233 out of 347 trough serum vancomycin concentrations were < 10 μg/mL. Although higher trough targets have not shown consistent associations with increased therapeutic efficacy, the guidelines have suggested they are effective in minimizing development of resistant strains, improving tissue penetration, and optimizing vancomycin pharmacodynamics [12, 26].

The current literature supports the favourable safety profile of vancomycin in neonates and young infants, with serum vancomycin trough concentrations being < 15 μg/mL [31, 32]. Thus, a validated PPK model providing a dosing algorithm of vancomycin in Chinese neonates and young infants, such as ours described herein, is significant for improving trough serum vancomycin concentrations as well as for strengthening physician confidence in the use of it.

In conclusion, a two-compartment PPK model was successfully established for vancomycin in Chinese neonates and young infants. Through the modelling, BBW and PNA were identified as significant covariates influencing the PK of vancomycin. The median CL and V values of vancomycin in Chinese neonates and young infants, as determined in this study, may be greater than those in neonates and young infants of other races, such as Caucasian, and further studies are necessary. Nonetheless, this new model was capable of accurately predicting PPK parameters of vancomycin and it was shown to be effective as a dosing algorithm of vancomycin in clinical treatment of Chinese neonates and young infants.

## MATERIALS AND METHODS

### Study design

This study used clinical data that had been collected routinely and was available in a de-identified format. The Ethics Committee of Children's Hospital of Chongqing Medical University determined no individual patient consent nor review by the Ethics Committee was required.

At stage 1 of the study, the Children's Hospital of Chongqing Medical University medical records database was searched retrospectively between November 2011 and December 2016. Patients were selected who received intravenous vancomycin that had been administered for a suspected or documented infection caused by Gram-positive bacteria and who had record of serum vancomycin concentration (detailed criteria listed below). Demographic data and routine clinical PK data were downloaded for the selected patients and used to establish a preliminary PPK model to investigate vancomycin PK parameters and to assess the parameters’ variability using the Phoenix^®^ NLME^TM^ modelling tool, version 1.3 (Certara L.P. Pharsight, St. Louis, MO, USA).

At stage 2, according to the preliminary PPK model, diagnostic scatter plots were used to evaluate the model's goodness-of-fit. A non-parametric bootstrap method and a visual predictive check (VPC) were then carried out for the internal evaluation of the final model. Data of additional patients, who had been admitted to Children's Hospital of Chongqing Medical University between January 2017 to June 2017, were then collected retrospectively for the external evaluation of the final model. Finally, the final model was preliminary validated with data of 2 young infants from Southwest Hospital of the Third Military Medical University.

### Patients and data collection

Criteria for study inclusion were: age of <60 days at the time of admission to hospital; suspected or confirmed bacterial infection that necessitated intravenously infusion of vancomycin as part of the standard of care, following the decision of the associate chief physician or chief physician; and, record of at least one serum vancomycin concentration that had been detected during the therapeutic process. Criteria for study exclusion were: receipt of renal replacement therapy; treatment with vancomycin for < 24 h; or, lack of demographic data.

Serum vancomycin concentrations were measured by our in-house Clinical Pharmacokinetic Service, using a fluorescence polarization immunoassay method that had been validated in terms of specificity, linearity, precision, accuracy and stability, and having a lower limit of quantification of 1 μg/mL. The following data were retrieved from the medical records of all patients included in the study: sex, gestational age (GA), postnatal age (PNA), postmenstrual age (PMA), birth body weight (BBW), body weight (BW), height (HT), body surface area [BSA; as m^2^=body weight(kg)^0.5378*^height(cm)^0.3964*^0.024265] [33], serum creatinine (SCr), SCr-based GFR (as mL/min/1.73 m^2^)=k^*^HT(cm)/SCr(mg/dL) [34], where *k* is 0.45 for full-term infants and 0.33 for preterm infants], blood urea nitrogen (BUN), alanine aminotransferase (ALT), albumin (ALB) and concomitant drug therapy. Additionally, data on vancomycin dose, administrationtime, blood sampling time and the determined concentrations of vancomycin were also collected. The Schwartz's formula [13] was chosen for calculation of GFR since it is currently the most commonly used in infants’ practice. In addition, we also collected the results of the patients’ pathogenic bacteria culture for providing some clinical reference.

### Model development

According to previously published studies, one- and two- compartment, open kinetic models with first-order elimination were examined for exploration of the mean structure during the modelling process. The basic PK parameters used in this study were volume of distribution for the central compartment (V1, l), clearance of the central compartment (CL, l/h) for the one-compartment model or for the two-compartment model with additional parameters defined as volume of distribution for the peripheral compartment (V2, l) and inter-compartmental clearance (Q, l/h).

Initial PK parameters were estimated by classic compartment models and non-compartment model using Phoenix^®^ WinNonlin 6.4. Exponential-error models were used to describe inter-individual variability of the basic PK parameters: *P_i_*=P_pop_^*^exp(*η_i_*), where *P_i_*is the individual parameter estimate of the *i*th subject, *P_pop_* is an estimate of the population mean of parameter *P* (on behalf of a basic PK parameter), and *η_i_* is the deviation from the population mean for the *i*th individual under the assumption that *η*'s are normally distributed with mean 0 and variance ω^2^.

In the process of model development, the candidate covariates were sex, GA, PNA (at vancomycin concentrations determined), PMA, BBW, BW, HT, BSA, SCr, GFR, BUN, ALT, ALB and concomitant drug therapy. Concomitant drug use only involved ∼ 20% of the studied patients. Continuous covariates were implemented using an allometric model with the following equation: *P*_i_= *P*_pop_^*^(*Cov*/*Cov*_median_)^dPdCov, where *P*_i_represents the individual parameter estimate of the *i*th subject, *P*_pop_represents the population parameter estimates, *Cov* is the covariate, and dPdCovis the exponent. Categorical covariates were implemented using the following equation: *P*_i_= *P*_pop_^*^exp (dPdCov^*^Cov), where *Cov* is a dummy variable that took on a value of 1 or 0.

The selection of covariates was determined using a forward-selection process and then a backward-elimination process (stepwise option in Phoenix^®^ NLME^TM^, version 1.3). The criterion for estimation of statistical significance was a reduction or increase in the value of objective function (OFV; −2log likelihood) between these models. During forward-selection, any covariate that reduced the OFV by > 6.635 [*p*<0.01, χ^2^ distribution with 1 degree of freedom (df)] was considered to be significant and added to the model. The full model was constructed with all statistically significant covariates included. The importance of each covariate was then re-evaluated by backward-elimination. Each covariate was independently removed from the model, by one at a time, to identify its relevance. An increase in the OFV of > 10.828 (*p*<0.001, χ^2^ distribution with 1 df) was required for confirmation.

The resulting model was termed the ‘final’ modeland included all significant covariates that could not be eliminated from the full model. Other diagnostic criteria for the retention of a covariate in the model were a reduction in unexplained inter-individual variability for the associated PK parameter, an improvement in the diagnostic scatter plots, and the 95% confidence interval (CI) estimated using standard errors (SEs) not including a zero value. The residual error was evaluated with additive, proportional, and combined residual error models, respectively.

### Model evaluation

#### Internal model evaluation

Goodness-of-fit was evaluated by using diagnostic scatter plots as follows: (a) observed (DV) versus population predicted concentrations (PRED); (b) DV versus individual predicted concentrations (IPRED); (c) conditional weighted residuals (CWRES) versus time (IVAR); (d) CWRES versus PRED; (e) quantile-quantile plot of the components of conditional weighted residuals. The stability of the final model was evaluated using the non-parametric bootstrap analysis (Bootstrap option in Phoenix® NLME^TM^, version 1.3) with 2,000 datasets, randomly sampled from the original dataset, with replacement. The values of estimated parameters, such as the medians and SEs from the bootstrap procedure, were compared with those estimated from the original dataset. The 95% CIs were obtained as the point estimate ±1.96^*^SE of the estimate.

The predictive performance of the final model was evaluated using the VPC method (VPC option in Phoenix® NLME^TM^, version 1.3). The VPC used Monte Carlo simulation to generate concentration-time profiles of 1,000 patients. Then, the DV concentration-time data were graphically superimposed on the median values and the 5th and 95th percentiles of the simulated concentration–time profiles. The model was deemed precise if the DV concentration data were approximately distributed within the 5th to 95th prediction interval.

#### External model evaluation

Estimates of the model were fixed during the process and drug concentrations of the patients enrolled for the external evaluation were predicted by calculation with the final model. The predictive performance of the model was evaluated by mean prediction error (MPE), mean absolute prediction error (MAE) and mean squared prediction error (MSPE), which were calculated by the following equations:

$$MPE=\frac{1}{n}\sum_{i=1}^{n}(c_{predi}-c_{obsi}),\;(n=1,2,...,i),$$

$$MAE=\frac{1}{n}\sum_{i=1}^{n}(|\;c_{predi}-c_{obsi}\;|),\;(n=1,2,...,i),$$

$$MSPE=\frac{1}{n}\sum_{i=1}^{n}{\left(c_{obsi}-c_{predi}\right)}^{2},\;(n=1,2,...,i),$$

where *C*_predi_ represents the predicted concentration of the *i*th subject and *C_obsi_* represents the DV concentration for the *i*th subject.
